# Supplementary material for: Comparative pathogenesis of different phylogroup I bat lyssaviruses in a standardized mouse model
Source: PLoS Negl Trop Dis. 2022 Jan 18;16(1):e0009845. doi: 10.1371/journal.pntd.0009845 (PMC8797209; doi:10.1371/journal.pntd.0009845)
Supplement: S1 Table — The reaction conditions were identical to those used for EBLV-1, BBLV and EBLV-2. (DOCX) [file pntd.0009845.s001.docx]

**S1 Table: Details of the primer and probe mix for GBLV. The reaction conditions were identical to those used for EBLV-1, BBLV and EBLV-2.**

| **Volume** | **Oligo** (Concentration) | **Sequence Primer/Probe** (5`- 3`) |
| --- | --- | --- |
| 20.0 µl | **GBLV_for**  (100 pmol/µl) | CCGGCGATTAGAGATCAAAAG |
| 20.0 µl | **GBLV_rev**  (100 pmol/µl) | CATTCCAGACAGAACGGAC |
| 5.0 µl | **GBLV_HEX probe** (100 pmol/µl) | HEX-CCTAGTATAACTCTTGGTAAGGCCCCAGAT-BHQ1 |
| 155.0 µl | 0.1 x TE (pH 8.0) |  |
| **200.0 µl** | **Primer-probe-mix** |  |
