# Supplementary material for: Comparative pathogenesis of different phylogroup I bat lyssaviruses in a standardized mouse model
Source: PLoS Negl Trop Dis. 2022 Jan 18;16(1):e0009845. doi: 10.1371/journal.pntd.0009845 (PMC8797209; doi:10.1371/journal.pntd.0009845)
Supplement: S2 Table — (DOCX) [file pntd.0009845.s002.docx]

**S2 Table: Clinical score sheet for mice, ranging from zero up to five.**

| **Score** | **Clinical Signs** | **Instructions / Humane Endpoints** |
| --- | --- | --- |
| **0** | - **Healthy/Normal** | **Daily observation** |
| **1** | - **Ruffled fur** - **Hunched back** | **Shorten observation intervals to 12h** |
| **2** | - **Slowed movements** - **Circular movements** - **Weight loss ≥ 15 %** | **Euthanasia within the next 8 hours maximum** |
| **3** | - **Tremor** - **Wobbly gait** - **Seizures** - **Weight loss ≥ 20** **%** | **Immediate Euthanasia** |
| **4** | - **Signs of paralysis or spasms** - **Weight loss ≥ 25** **%** | **Immediate Euthanasia** |
| **5** | - **Coma/Death** | **Immediate Euthanasia** |
