# Supplementary figures and images for: Comparative pathogenesis of different phylogroup I bat lyssaviruses in a standardized mouse model
Source: PLoS Negl Trop Dis. 2022 Jan 18;16(1):e0009845. doi: 10.1371/journal.pntd.0009845 (PMC8797209; doi:10.1371/journal.pntd.0009845)

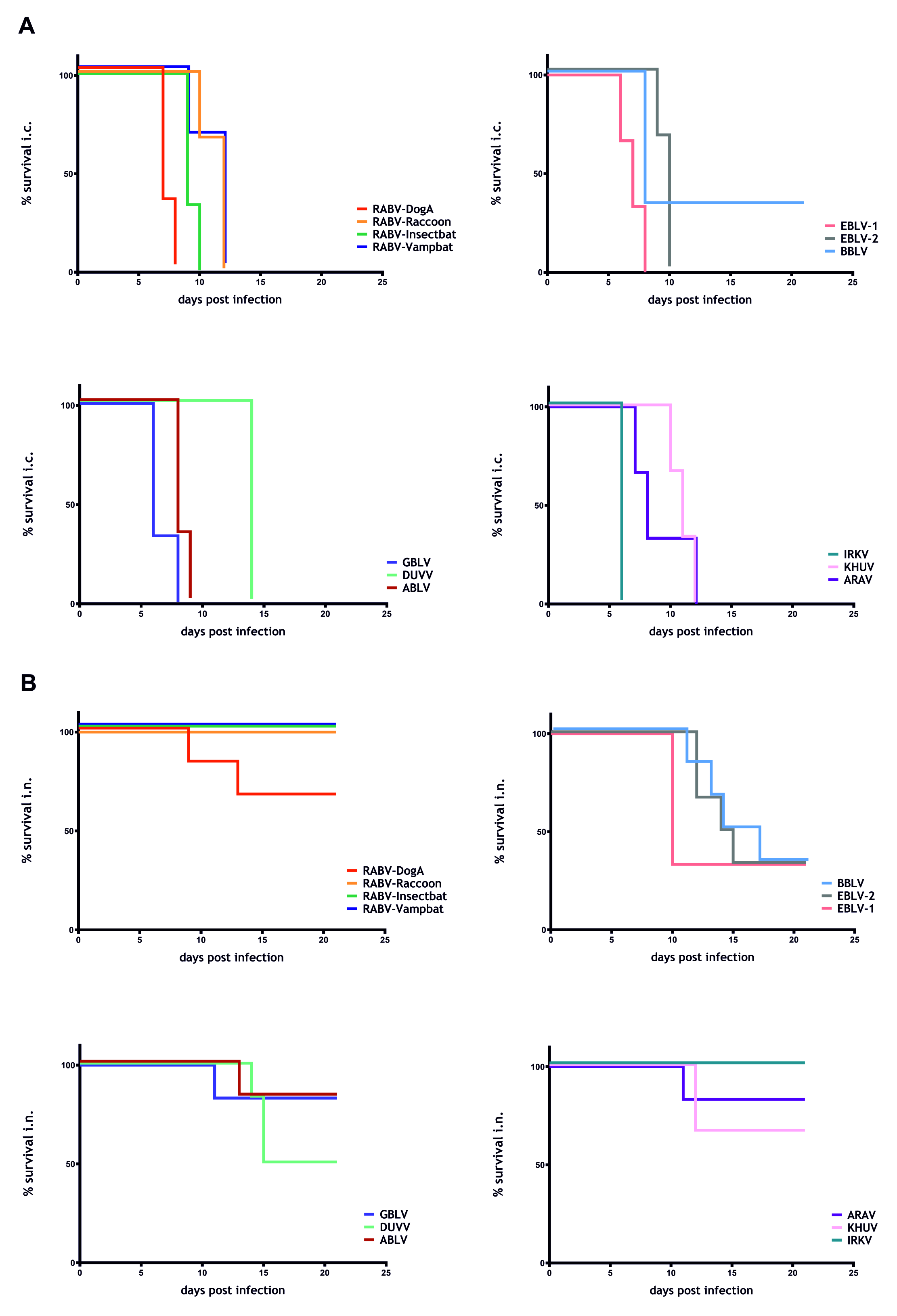

Supplement: S1 Fig — Kaplan-Maier survival plots of the individual isolates following i.c. infection (six Balb/c mice were inoculated per group) (A) and i.n. infection (three Balb/c mice were inoculated group) (B). Mock-infected control mice did not develop any clinical signs and, hence, were omitted for better visualization. (TIF) [file pntd.0009845.s003.tif]
